# Supplementary material for: Hyperstretching DNA
Source: Nat Commun. 2017 Dec 19;8:2197. doi: 10.1038/s41467-017-02396-1 (PMC5736724; doi:10.1038/s41467-017-02396-1)
Supplement: Supplementary file 1 — Supplementary Information [file 41467_2017_2396_MOESM1_ESM.pdf]

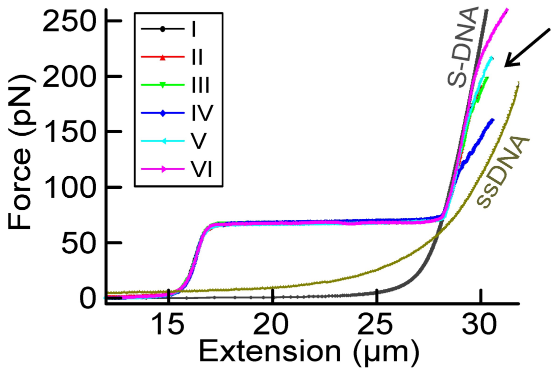

Supplementary Figure 1: Peeling hampers the use of force-extension curves for the identification of HS-DNA formation.

We tested different salt conditions in the buffer (150-1000 mM NaCl, 0-50 mM $MgCl_2$) in order to obtain maximum dsDNA force stability, in particular, to minimize peeling. We eventually settled for 1000 mM NaCl, since results indicated the least propensity for peeling; nevertheless, even here peeling becomes significant at elevated forces. Supplementary Fig. 1 shows six forward stretching curves obtained in high salt which display excellent overlap up to 100 pN force; for comparison, we also show an experimental curve for ssDNA (dark yellow), as well as the theoretically derived curve for S-DNA (dark gray, see details below). It becomes obvious that, at forces larger than 100 pN, the curves start to increasingly diverge from the course expected for S-DNA and deviate to longer extensions seen for single-stranded DNA. The rather irregular appearance in this high force regime (arrow), and the force range around 150 pN is in line with previous reports for peeling [1]. A theoretical model was used for S-DNA because we can not obtain this curve experimentally. The dark grey curve for S-DNA displayed in Supplementary Fig. 1 is generated from a model proposed by Zhang *et al.* [2], using model parameters bp-contour length = 0.585 nm, persistence length = 13 nm, stretch modulus = 3200 pN, similar to those reported by Zhang *et al.* Note that the DNA breaks at the highest forces displayed, therefore, no retraction hysteresis caused by peeling is shown.

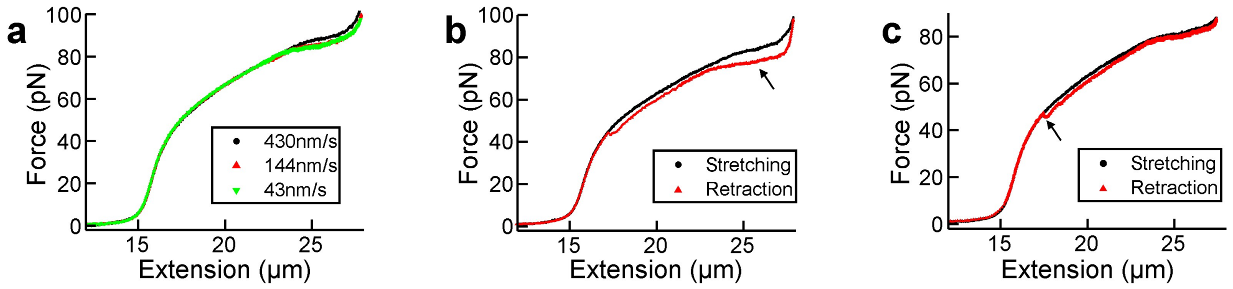

Supplementary Figure 2: Determining equilibrium conditions for the recording or force-extension curves.

Two different approaches show that intercalator binding to the DNA and structural changes occurring within the DNA are in equilibrium while manipulating the DNA extension. In the first method, we record forward stretching curves at different speeds and look for systematic changes in the force-extension curves. In Supplementary Fig. 2a, recorded for a YO-PRO concentration of 667 nM, we find that at 430 nm s^{-1} stretch speed (black curves), the overstretching region (> 23.5 μm extension) shows a significantly higher force plateau compared to a curve recorded at 144 nm s^{-1} (red). Changes in the overstretching plateau have previously been shown to be indicators of equilibrium conditions for intercalator binding [3]. Since a further reduction in stretch speed to 43 nm s^{-1} (green) does not result in any further changes to the curve such that green and red stretch curves overlap, we deduce thus that in the case of 667 nM YO-PRO, stretching curves recorded at a stretch speed of 144 nm s^{-1} and slower are in equilibrium. This finding is further verified by comparing a force extension curve below equilibrium speed (black curve in Supplementary Fig. 2b/c), stretched until the end of the overstretching (OS-) region (extension > 27 pN, characterized by a much sharper force increase), with the corresponding retraction curve obtained at the same speed. The lack of significant hysteresis between forward- and retraction curve indicates equilibrium binding conditions. Indeed, while extending and retracting at 430 nm s^{-1} (2b) shows significant hysteresis in the OS region (arrow), this is not the case at 144 nm s^{-1} (c). Note that a slight hysteresis in the retraction curve in Supplementary Fig. 2b (red) is still observed at 144 nm s^{-1}; Because this slight hysteretic effect exhibits at $\sim$ 45 pN in a rather sharp force spike back to the force of the forward stretching curve (arrow), we attribute it to a small section ($<$1.5 kbp, as estimated from the lateral curve shift) of ssDNA which unpeeled from one dsDNA-end during the slow OS transition [4], and which experiences a sudden reannealing event at forces well below the melting force of bare DNA ($\sim$65 pN). These unpeeling and reannealing events can be clearly observed in fluorescence and have been excluded from fluorescence intensity analysis (Supplementary Fig. 3).

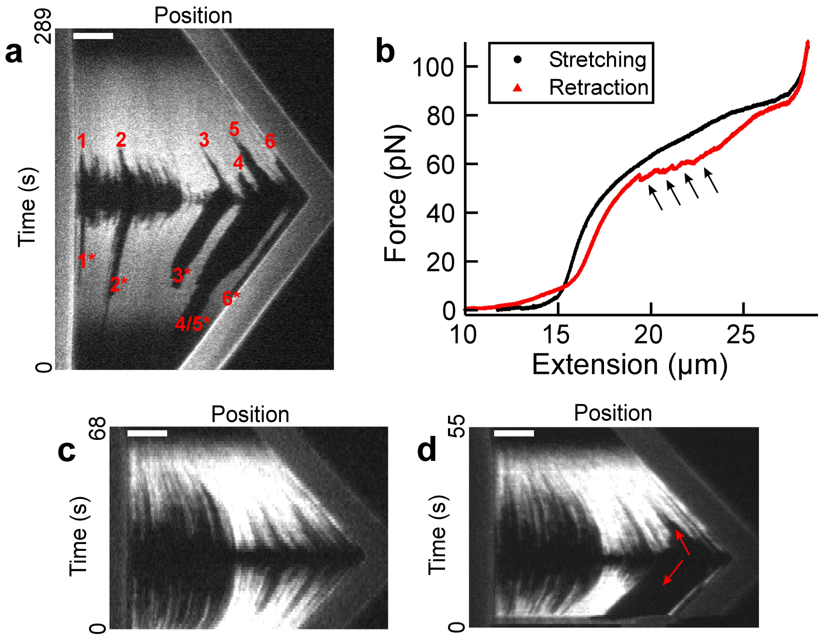

Supplementary Figure 3: Differentiation between S-DNA formation and peeling (from nicks).

For the observation of the transition to both S- and HS-DNA, it is a prerequisite that the DNA does not undergo peeling to form single-stranded DNA as a competing pathway [5]. It is well established that DNA melting is favoured in the case of nicks (breaks in the backbone) being present which allows one strand to peel off [1, 6]. The application of fluorescent intercalators allows us to unambiguously detect the presence and extent of peeling occurring from nicks and DNA ends. Supplementary Fig. 3a/b displays the fluorescence kymograph of a DNA stretched until the end of the OS-transition and then retracted (a), as well as the corresponding force extension curve (b). The occurrence of local peeling can therefore be distinguished by the appearance of dark sections of DNA (numbered 1-6) in the forward stretching section of the OS kymographs (a). These sections display a large hysteresis (i.e., reannealing at an extension that is shorter than the extension where peeling started) in the corresponding retraction curve (indicated by the same number with a star). The delayed reappearance of B-DNA compared to S-DNA is easily explained by the formation of hairpin structures in the peeled single-stranded sections which need to be broken before reannealing can occur [4]. This hysteresis is also visible in the force-extension curves (b) and - in difference to intercalation-related hysteresis in the OS region (Supplementary Fig. 2c) - results in inhomogeneous, sudden force spikes in the retraction curve (arrows), caused by the resolution of hairpin structures in the peeled-off strand. On the other hand, the high reproducibility of the OS fluorescence pattern enables us to directly identify peeling sections here even without the necessity of checking for hysteresis. For demonstration, Supplementary Fig. 3c/d shows kymographs for two subsequent stretching/retraction cycles of the same DNA molecule, measured in the presence of the mono-intercalator Sytox Orange. The first curve (c) shows a fluorescence pattern which is identical for stretching and retraction section, indicating that there is no stretching hysteresis. However, the subsequent stretch/retraction cycle (d) reveals the appearance of a dark gap in stretching section which has become much larger in the retraction section (arrows). This gap is caused by peeling from a nick likely caused by photochemically induced damage on the DNA which is a well-known feature of intercalator fluorescence [7]. Thus, that nick-free DNAs produce a robust and reproducible pattern (Fig. 5) means that the dark gap in the forward stretching section which is not found for other DNA molecules is sufficient alone to identify the occurrence of DNA-peeling. While many nick-free curves do show a peeling section occurring from one of the free DNA-ends (e.g., see section Nr. 6/6* in Supplementary Fig. 3, and Supplementary Fig. 4), the section is typically quite small ($< 5\%$ of full contour length), thus does not have a significant effect on the general conclusions regarding HS-DNA. The scale bar in a, c and d has a length of 5 μm.

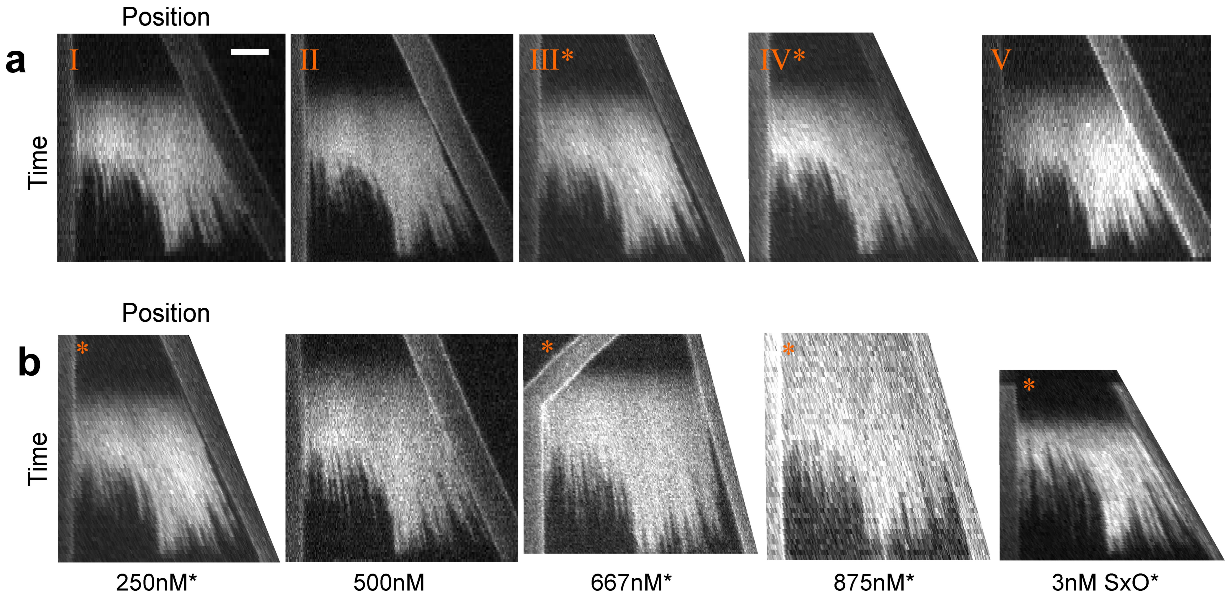

Supplementary Figure 4: The OS-darkening pattern is highly reproducible.

Supplementary Fig. 4a displays kymographs (length/time axes horizontal/vertical, respectively) obtained for different DNA molecules at 250 nM YO-PRO (a) and at different dye concentrations of YO-PRO (concentration value given), as well as for the mono-intercalator SxO (b). The observation that identical patterns are observed virtually independent of intercalator concentration as well as dye supports the idea that the observed pattern is governed by the DNA structure itself and not imposed by the intercalating molecules.

The scale bar shown in kymograph I has a length of 5 μm and applies to all other curves. Kymographs are obtained at stretching speeds ranging from 140-700 nm s^{-1} and are aligned to allow for best appreciation of the reproducibility of the pattern. The stretching rate of the molecule does not influence the pattern, as long as stretching is slow enough to allow the intercalator to detach, but fast enough to minimize peeling (for the same reason, the time scales of the kymographs is not relevant, and therefore omitted here for clarity). Note also that curves marked with a star display in the raw data OS patterns which are inverted compared to the other kymographs, but have been converted in order to display identical pattern orientation. The occurrence of two different patterns simply reflects the fact that we cannot control the DNA sequence orientation in our approach, therefore both patterns are observed with roughly equal probability.

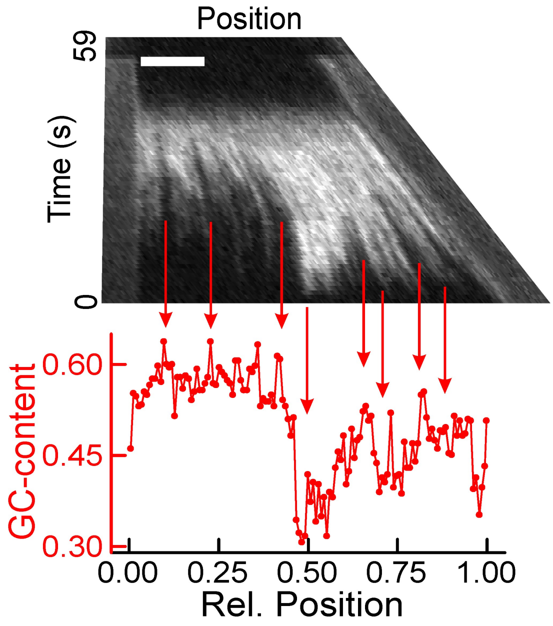

Supplementary Figure 5: The OS-darkening pattern correlates with the AT/GC-content of the DNA.

We propose here that both the first B/S-, as well as the second S/HS-DNA transition is not only force, but also dependent on the AT/GC-content of the stretched sequence. In particular, we propose that in AT-richer sections the first (B- to S-DNA) transition occurs at higher, but the second (S- to HS-DNA) transition at lower forces compared to sections with higher GC-content. This correlation can be particularly well appreciated by displaying the GC-content directly below the OS-kymograph (Supplementary Fig. 5). Then it becomes obvious that early occurrence of dark stripes (high) correlates well with peaks whereas long lasting bright sections (low arrows) are observed in regions of dips in the GC-content map. Note that the only exception is the GC-low segment very close to the right DNA end which correlates with the appearance of a dark stripe. This, however, is most likely caused by a small peeling front occurring from this side, since AT-rich sequences are much more prone to peeling compared to GC-rich sequences. The scale bar in the kymograph has a length of 5 μm.

Supplementary Note 1: Model parameters

Our model contains 8 free parameters: l_0, γ_1, ε_1, λ, γ_2, η, ε_2 and δ (see also the Methods section in the main text). Here we describe the fitting procedure we use to obtain these parameters from experimental data, and justify this large number of parameters by describing for each of them which specific feature of the force-extension curve they control. We use the following fitting procedure:

1. l_0 determines the DNA extension of B-DNA, γ_1 determines the relative length of S-DNA compared to B-DNA, ε_1 determines the force, at zero intercalator concentration, required for overstretching and λ the cooperativity of the overstretching transition. These four parameters are properties of DNA only, so we obtain their values from a fit, using equation (S6), on the zero-concentration curve.

2. γ_2 is the relative length of intercalated DNA compared to B-DNA, and determines the extension of hyper-stretched (HS-) DNA. We fix its value to 2.0 based on the known local extension of DNA from 0.34 nm per bp to 0.68 nm per bp [8].

3. η controls the steepness of the shifted overstretching transition at intercalator concentrations 250-875 nM. We estimate the value of η by comparing the steepness of the shifted overstretching transition in the theoretical curves with that in the experimental data in the concentration range from 500 to 875 nM.

4. Finally, we perform a single global fit, using the full model given in equation (S6), on the force-extension curves in the presence of 250 nM, 500 nM, 583 nM, 667 nM and 750 nM YO-PRO to determine the values of ε_2 and δ. Here, ε_2 determines the shift of the force required for overstretching as a function of intercalator concentration; see also Fig. 4 in the main text. δ determines the concentration at which the transition from B-DNA to overstretched DNA is replaced by a gradual transition from B-DNA to intercalated DNA (feature 3).

We apply this fitting procedure to the data in Fig. 1 in the main text to obtain the model parameters for YO-PRO, and to the data by Vladescu and coworkers [9] to obtain them for EtBr. For YO-PRO we find $l_0 = 0.33\,\text{nm}$, $\gamma_1 = 1.8$, $\varepsilon_1 = 3.7\,k_BT$, $\lambda = 3.0\,k_BT$, $\gamma_2 = 2.0$, $\eta = 3.5\,k_BT$, $\varepsilon_2 = -13\,k_BT$ and $\delta = 1.5\,k_BT$. For EtBr we find $l_0 = 0.34\,\text{nm}$, $\gamma_1 = 1.7$, $\varepsilon_1 = 3.2\,k_BT$, $\lambda = 3.4\,k_BT$, $\gamma_2 = 2.0$, $\eta = 5.6\,k_BT$, $\varepsilon_2 = -18\,k_BT$ and $\delta = 3.4\,k_BT$.

Finally, we predict that the steepness of the hyperstretching transition from overstretched DNA to hyperstretched DNA (feature H in Fig. 2 in the main text) is determined by a competition between the parameters η and δ. We predict that the cooperativity, i.e., the effective energy penalty for neighbouring intercalated and overstretched base pairs compared to those of equal base pairs, of this transition is given by $\eta - \frac{1}{2}\delta$. Using the parameter values given above we expect the hyperstretching transition to be cooperative for both YO-PRO and EtBr.

Supplementary Note 2: Full expressions for the state fractions

The fractions ϕ_i of base pairs in the different states i ($i = 0, 1, 2$) are derived by applying equation (S4). The rather unwieldy, but completely analytic results are given by

$$\phi_i = \frac{X'_{1,i} + \left(X'_{3,i} + \frac{X_3 X'_{3,i} - 6X_2^2 X'_{2,i}}{2Y_1}\right)\left(\frac{1}{6Y_2^2} - \frac{X_2}{6Y_2^4}\right) + \frac{X'_{2,i}}{Y_2}}{X_1 + Y_2 + \frac{X_2}{Y_2}} x_i, \tag{S1}$$

where Y_i, X_i, and $X'_i = \frac{\partial X_i}{\partial \chi}$ are given by:

$$Y_1 = \sqrt{X_3^2/4 - X_2^3} \qquad\qquad Y_2 = \sqrt[3]{X_3/2 + Y_1}$$

$$X_1 = x_0 + x_1 + x_2$$
$$X_2 = X_1^2 - 3\left[E_{01}x_0x_1 + E_{02}x_0x_2 + E_{12}x_1x_2\right]$$
$$X_3 = 2X_1^3 - 9\left[E_{01}(x_0^2x_1 + x_0x_1^2) + E_{02}(x_0^2x_2 + x_0x_2^2) + E_{12}(x_1^2x_2 + x_1x_2^2) + E_{012}x_0x_1x_2\right]$$

$$X_1' = x_0' + x_1' + x_2'$$
$$X_2' = 2X_1X_1' - 3\left[E_{01}(x_0'x_1 + x_1'x_0) + E_{02}(x_0'x_2 + x_2'x_0) + E_{12}(x_1'x_2 + x_2'x_1)\right]$$
$$X_3' = 6X_1^2X_1' - 9\Big\{E_{01}[x_0'x_1^2 + 2(x_0' + x_1')x_0x_1 + x_1'x_0^2] + E_{02}[x_0'x_2^2 + 2(x_0' + x_2')x_0x_2 + x_2'x_0^2]$$
$$+ E_{12}[x_1'x_2^2 + 2(x_1' + x_2')x_1x_2 + x_2'x_1^2] + E_{012}(x_0'x_1x_2 + x_1'x_0x_2 + x_2'x_0x_1)\Big\}$$

Here, all E and lower case x_i and x_i' are defined as

$$x_0 = \sinh(\chi), \qquad x_1 = e^{-\epsilon_1}\sinh(\gamma_1\chi)/\gamma_1 \qquad x_2 = e^{-\epsilon_2+\mu-\delta}\sinh(\gamma_2\chi)/\gamma_2$$
$$x_0' = \cosh(\chi), \qquad x_1' = e^{-\epsilon_1}\cosh(\gamma_1\chi) \qquad x_2' = e^{-\epsilon_2+\mu-\delta}\cosh(\gamma_2\chi)$$
$$E_{01} = 1 - e^{-2\lambda} \qquad E_{02} = 1 - e^{\delta} \qquad E_{12} = 1 - e^{\delta-2\eta}$$
$$E_{012} = 2e^{-2\lambda} + 2e^{\delta} + 2e^{\delta-2\eta} - 6e^{\delta-\lambda-\eta}$$

where ε_1, ε_2, μ, λ, η, δ, γ_1 and γ_2 are defined in the main text. Finally, $X_{j,i}' = \frac{\partial X_j'}{\partial x_i'}$ are given by

$$X_{1,i}' = 1$$
$$X_{2,0}' = 2X_1 - 3(E_{01}x_1 + E_{02}x_2)$$
$$X_{2,1}' = 2X_1 - 3(E_{01}x_0 + E_{12}x_2)$$
$$X_{2,2}' = 2X_1 - 3(E_{02}x_0 + E_{12}x_1)$$
$$X_{3,0}' = 6X_1^2 - 9\left[E_{01}(x_1^2 + 2x_0x_1) + E_{02}(x_2^2 + 2x_0x_2) + E_{012}x_1x_2\right]$$
$$X_{3,1}' = 6X_1^2 - 9\left[E_{01}(x_0^2 + 2x_0x_1) + E_{12}(x_2^2 + 2x_1x_2) + E_{012}x_0x_2\right]$$
$$X_{3,2}' = 6X_1^2 - 9\left[E_{02}(x_0^2 + 2x_0x_2) + E_{12}(x_1^2 + 2x_1x_2) + E_{012}x_0x_1\right].$$

Supplementary Note 3: Model derivations

In the limit of zero persistence and backbone stretching, we obtain the extension of our chain z as a function of the stretching force f by

$$\left\langle \frac{z}{L_0} \right\rangle = \frac{k_BT}{L_0}\frac{\partial}{\partial f}\ln Z, \tag{S2}$$

where Z is the partition function defined in equation (3) in the Methods section of the main text, $L_0 = Nl_0$ is the contour length of B-DNA, k_B is Boltzmann's constant, T is the absolute temperature, and $z/L_0 = 1$ corresponds to a DNA extension of l_0 per basepair. The result can be written as

$$\left\langle \frac{z}{L_0} \right\rangle = \phi_0(f,C)\,\mathcal{L}(\beta f l_0) + \phi_1(f,C)\,\gamma_1\,\mathcal{L}(\beta f\gamma_1 l_0) + \phi_2(f,C)\,\gamma_2\,\mathcal{L}(\beta f\gamma_2 l_0), \tag{S3}$$

where $\beta = \frac{1}{k_BT}$ and $\phi_0(f,C)$, $\phi_1(f,C)$ and $\phi_2(f,C)$ are the fractions of base pairs in the B-DNA, overstretched and intercalated states that depend on both stretching force f and intercalator concentration C. $\mathcal{L}(x) = \coth(x) - \frac{1}{x}$ is the Langevin function and we recognize the freely-jointed chain force-extension curves [10] in $\mathcal{L}(\beta f l_0)$, $\gamma_1\mathcal{L}(\beta f\gamma_1 l_0)$ and $\gamma_2\mathcal{L}(\beta f\gamma_2 l_0)$ for chains with Kuhn lengths l_0 (B-DNA), $\gamma_1 l_0$ (overstretched DNA) and $\gamma_2 l_0$ (intercalated DNA).

To put this in words, the force-extension relation in the limit of vanishing persistence and backbone stretching can be written as a superposition of the force-extension relations of the freely-jointed chains of the three states of our

model, with the fractions of base pairs in the different states as weights. All details of the model are thus contained in the fractions, which are found by

$$\phi_2(f, C) = \langle \delta_{S_i,2} \rangle \ = \ \frac{\partial}{\partial \mu} \ln Z, \tag{S4a}$$

$$\phi_1(f, C) = \langle \delta_{S_i,1} \rangle \ = \ -\frac{\partial}{\partial \varepsilon_1} \ln Z, \tag{S4b}$$

$$\phi_0(f, C) = \langle \delta_{S_i,0} \rangle \ = \ 1 - \phi_1 - \phi_2. \tag{S4c}$$

The full expressions for the three fractions are given in Supplementary Note 2. We use this observation to include the principal effects of persistence and backbone stretching in our model. The idea is to replace the freely-jointed chain extensions in equation (S3) with extensions given by the extensible worm-like chain (WLC) model [11, 12] for each of the three states. We use the approximate force-extension relation found by Odijk [13], which is valid in the force regime $f \gtrsim 10$ pN,

$$\left\langle \frac{z}{L_0} \right\rangle = 1 - \frac{1}{2\sqrt{\beta f P}} + \frac{f}{K}, \tag{S5}$$

where P is the persistence length and K the stretching modulus of the worm-like chain. The corrected force-extension relation of our 3-state model is then given by

$$\left\langle \frac{z}{L_0} \right\rangle = \phi_0(f, C) \left(1 - \frac{1}{2\sqrt{\beta f P_0}} + \frac{f}{K_0}\right) + \phi_1(f, C)\, \gamma_1 \left(1 - \frac{1}{2\sqrt{\beta f P_1}} + \frac{f}{K_1}\right) + \phi_2(f, C)\, \gamma_2 \left(1 - \frac{1}{2\sqrt{\beta f P_2}} + \frac{f}{K_2}\right), \tag{S6}$$

where $P_0 = 50$ nm and $K_0 = 1000$ pN [14] are the persistence length and stretching modulus of B-DNA and $P_1 = 13$ nm and $K_1 = 2700$ nm [2] those of overstretched DNA. What numbers to use for the persistence length P_2 and stretching modulus K_2 of intercalated DNA is, however, less clear because these have, as far as we know, not been reported for fully intercalated DNA. Some authors have fitted the WLC model predictions to experimental force-extension relations [15, 16], and found lower persistence lengths compared to bare DNA. However, these efforts assume a constant coverage of intercalators, which is not the case for our equilibrium force-extension relations. Biebricher *et al.* [3] observed that intercalators merely change the contour length of DNA, while the persistence length is mostly unaffected by the presence of intercalators. Therefore we take the persistence length of intercalated DNA to be the same as that of B-DNA, $P_2 = 50$ nm. Even less is known about the stretching modulus of (fully) intercalated DNA. Since we believe intercalated DNA segments are stiffer than B-DNA, we take the stretching modulus ten times as large, $S_2 = 10000$ pN.

The overstretching force $f_{\rm os}$ is found, for a set of given model parameters ε_1, ε_2, γ_1 and γ_2, and given concentration C, by numerically solving $\langle z/L_0 \rangle = 1.6$, using equation (S6). By varying the concentration C and the values of the model parameters in this procedure, the approximate relation between the overstretching force and the concentration, as given in equation (1) in the main text, is found numerically.

Supplementary References

[1] Cocco, S., Yan, J., Léger, J.-F., Chatenay, D. & Marko, J. F. Overstretching and force-driven strand separation of double-helix DNA. *Phys. Rev. E* **70**, 011910 (2004).

[2] Zhang, X. *et al.* Revealing the competition between peeled ssDNA, melting bubbles, and S-DNA during DNA overstretching by single-molecule calorimetry. *Proc. Natl Acad. Sci. USA* **110**, 3865–3870 (2013).

[3] Biebricher, A. S. *et al.* The impact of DNA intercalators on DNA and DNA-processing enzymes elucidated through force-dependent binding kinetics. *Nat. Commun.* **6** (2015).

[4] Gross, P. *et al.* Quantifying how DNA stretches, melts and changes twist under tension. *Nat. Phys.* **7**, 731–736 (2011).

[5] Paik, D. H. & Perkins, T. T. Overstretching DNA at 65 pN does not require peeling from free ends or nicks. *J. Am. Chem. Soc.* **133**, 3219–3221 (2011).

[6] King, G. A. *et al.* Revealing the competition between peeled ssDNA, melting bubbles, and S-DNA during DNA overstretching using fluorescence microscopy. *Proc. Natl Acad. Sci. USA* **110**, 3859–3864 (2013).

[7] Ihmels, H. & Otto, D. Intercalation of organic dye molecules into double-stranded DNA–general principles and recent developments. In *Supermolecular dye chemistry*, 161–204 (Springer, 2005).
[8] Berman, H. M. & Young, P. R. The interaction of intercalating drugs with nucleic acids. *Annu. Rev. Biophys. Bioeng.* **10**, 87–114 (1981).
[9] Vladescu, I. D., McCauley, M. J., Rouzina, I. & Williams, M. C. Mapping the phase diagram of single DNA molecule force-induced melting in the presence of ethidium. *Phys. Rev. Lett.* **95**, 158102 (2005).
[10] Flory, P. *Statistical Mechanics of Chain Molecules* (Interscience, 1969).
[11] Bustamante, C., Smith, S. B., Liphardt, J. & Smith, D. Single-molecule studies of DNA mechanics. *Curr. Opin. Struct. Biol.* **10**, 279 – 285 (2000).
[12] Wang, M. D., Yin, H., Landick, R., Gelles, J. & Block, S. M. Stretching DNA with optical tweezers. *Biophys. J.* **72**, 1335–1346 (1997).
[13] Odijk, T. Stiff chains and filaments under tension. *Macromolecules* **28**, 7016–7018 (1995).
[14] Storm, C. & Nelson, P. C. Theory of high-force DNA stretching and overstretching. *Phys. Rev. E* **67**, 051906 (2003).
[15] Vladescu, I. D., McCauley, M. J., Nuñez, M. E., Rouzina, I. & Williams, M. C. Quantifying force-dependent and zero-force DNA intercalation by single-molecule stretching. *Nat. Methods* **4**, 517–522 (2007).
[16] Husale, S., Grange, W. & Hegner, M. DNA mechanics affected by small DNA interacting ligands. *Single Molecules* **3**, 91–96 (2002).
